# Supplementary material for: The genome sequence of the False Heath Fritillary, Melitaea diamina (Lang, 1789) (Lepidoptera: Nymphalidae)
Source: Wellcome Open Res. 2025 Aug 11;10:437. [Version 1] doi: 10.12688/wellcomeopenres.24719.1 (PMC12521902; doi:10.12688/wellcomeopenres.24719.1)
Supplement: Peer review history

# Reviewer report: Reviewer response for version 1

**DOI:** 10.21956/wellcomeopenres.27235.r132981

**Published:** 2025-10-14

**Authors:** Stuart J.E. Baird

**Affiliations:** 1 Institute of Veretebrate Biology, Academy of Sciences of the Czech Republic, Národní, Staré Město, Czech Republic

The manuscript meets the genome note criteria. I find the layout and format of these notes very beautiful and pleasing.

 The motivation “…sequenced as part of Project Psyche” is terse. A link to the Project Psyche would help, maybe some mention of its goals.

 The Figure 2 heatmap has no key for colours. The reader must assume darker colours mean more “Hi-C contact” but are left with no quantification. How big is the difference in “Hi-C contact” between brown and yellow, and what does this mean? White is none, and brown is 'complete'?

Are sufficient details of methods and materials provided to allow replication by others?

Yes

Is the rationale for creating the dataset(s) clearly described?

Partly

Are the datasets clearly presented in a useable and accessible format?

Yes

Are the protocols appropriate and is the work technically sound?

Yes

Reviewer Expertise:

Admixture genomics

I confirm that I have read this submission and believe that I have an appropriate level of expertise to confirm that it is of an acceptable scientific standard.

# Reviewer report: Reviewer response for version 1

**DOI:** 10.21956/wellcomeopenres.27235.r132986

**Published:** 2025-10-09

**Authors:** Wei Lu

**Affiliations:** 1 The University of Chicago, Chicago, Illinois, USA

Wymann et al. report a haplotype-resolved, chromosome-level genome assembly of the False Heath Fritillary (Melitaea diamina), including the Z chromosome. The assembly spans approximately 578 Mb, with a contig N50 of 10.52 Mb, scaffold N50 of 19.75 Mb, and BUSCO completeness of about 98.5%, representing a high-quality reference genome. The addition of chromosome painting based on Merian elements provides useful comparative context. The methods are clearly described, and the data are readily accessible. This high-quality genome assembly will be valuable for future comparative and evolutionary genomic studies.

 Comments:

 1. In the Hi-C contact map, there appear to be strong off-diagonal interaction signals between chr22 and chr8 and also between chr22 and chr24. This may suggest potential mis-joins or local assembly issues, although repeats could also produce similar patterns. Clarification would be helpful.

 Minor comments:

 2. In the introduction, the manuscript notes that multiple color morphs exist. It would be useful to specify which morph the sequenced individual represents.

 3. The meaning of the EBP metric “7.C.Q66” could be briefly explained for clarity.

 4. The manuscript states that chromosome numbering was assigned by size. It would be helpful to clarify whether this naming convention aligns with other Melitaea species to facilitate cross-species comparisons.

Are sufficient details of methods and materials provided to allow replication by others?

Yes

Is the rationale for creating the dataset(s) clearly described?

Yes

Are the datasets clearly presented in a useable and accessible format?

Yes

Are the protocols appropriate and is the work technically sound?

Yes

Reviewer Expertise:

genomics, single-cell transcriptomics, developmental biology

I confirm that I have read this submission and believe that I have an appropriate level of expertise to confirm that it is of an acceptable scientific standard.
